# Dietary ellagic acid ameliorated *Clostridium perfringens*-induced subclinical necrotic enteritis in broilers via regulating inflammation and cecal microbiota

**DOI:** 10.1186/s40104-022-00694-3

**Published:** 2022-04-19

**Authors:** Yu Tang, Xinyue Zhang, Yanan Wang, Yongpeng Guo, Peiqi Zhu, Guiguan Li, Jianyun Zhang, Qiugang Ma, Lihong Zhao

**Affiliations:** 1 State Key Laboratory of Animal Nutrition, Poultry Nutrition and Feed Technology Innovation Team, College of Animal Science and Technology, China Agricultural University, No. 2. West Road Yuanming yuan, Beijing, 100193 People’s Republic of China; 2 Jiangsu Lihua animal husbandry Co., Ltd. No. 500, Hexi Village, Luxi Village Committee, Niutang Town, Wujin District, Changzhou City, Jiangsu Province 213168 People’s Republic of China; 3 COFCO feed Co., Ltd, 4th Floor, No. 6, Nandaan Hutong, Xicheng District, Beijing, 100193 People’s Republic of China

**Keywords:** Broiler, *Clostridium perfringens*, Ellagic acid, Intestinal microbiota, Subclinical necrotic enteritis

## Abstract

**Background:**

Subclinical necrotic enteritis (SNE), a common intestinal disease of broiler caused by *Clostridium perfringens*, could reduce production performance of broilers by chronic intestinal damage and poor absorption of nutrients. Ellagic acid (EA) has been reported to present antioxidant and anti-inflammatory properties on human and animals in many aspects. This study was conducted to evaluate the effect and mechanism of EA in relieving SNE in broilers induced by *C. perfringens*.

**Results:**

*C. perfringens* challenge decreased body weight (BW), average daily gain (ADG), jejunal villi height/crypt depth (V/C) ratio, the activity of catalase (CAT) and the mRNA expression of zonula occludens 1 (*ZO-1*) in jejunal mucosa of broilers. While feed conversion ratios (FCR), jejunal crypt depth (CD), the activities of myeloperoxidase (MPO) and diamine oxidase (DAO), as well as the concentrations of interleukin 6 (IL-6), C-reactive protein (CRP) and procalcitonin (PCT) in serum, the activities of inducible nitric oxide synthase (iNOS) and lysozyme (LZM), the concentration of malondialdehyde (MDA), and the mRNA expressions of claudin-2, *TNF-α*, *IL-1β*, *TLR-4*, *TLR-2*, *NF-κB*, *JAK3*, *STAT6* and *iNOS* in jejunal mucosa of broilers were increased by *C. perfringens* challenge. Dietary EA supplement relieved these adverse effects, and heightened jejunal villi height (VH), the concentration of D-xylose in plasma, activity of superoxide dismutase (SOD), and the mRNA expression of occludin in jejunal mucosa of broilers. The alpha diversity of cecal microbiota indicated that dietary EA supplement increased observed species and Shannon index. *C. perfringens* challenge increased the relative abundance of Firmicutes and decreased the relative abundance of Desulfobacterota in cecal microbiota. EA increased the relative abundance of Firmicutes in cecal microbiota. LEfSe analysis showed that *C. perfringens* challenge triggered the imbalance of cecal microbiota in broilers, dietary EA supplementation led to a small beneficial effect on microbiota, while the simultaneous effect of them seemed to stimulate the immune function of broilers by improving the microbiota balance.

**Conclusions:**

Dietary EA ameliorated *C. perfringens*-induced SNE in broilers via regulating jejunal inflammation signaling pathways TLR/NF-κB and JAK3/STAT6, relieving jejunal oxidative stress and balancing cecal microbiota to inhibit intestinal barrier damage, prevent systemic inflammatory response and improve nutrient absorption capacity, finally protect and enhance growth performance of broilers.

**Supplementary Information:**

The online version contains supplementary material available at 10.1186/s40104-022-00694-3.

## Introduction

Necrotic enteritis (NE) is a common inflammatory disease of small intestine caused by *Clostridium perfringens*. NE poses an important threat to various animals, including chickens, pigs, sheep and goats. *C. perfringens* is a spore-forming, anaerobic, gram-positive bacterium and an opportunistic pathogen found in environment and the intestinal microbiota of animals [1]. *C. perfringens* strains vary significantly in toxin production and have been divided into types A-G based on the presence of encoding genes for alpha (CPA), beta (CPB), epsilon (ETX), iota (ITX), NetB and CPE toxins [2]. NE, mainly caused by type A *C. perfringens*, leads to economic losses of $6 billion annually in the global poultry industry in poultry [3].

NE usually occurs in broiler at 2- to 6-week-old, and was divided into acute clinical NE and subclinical NE (SNE) [4]. Acute clinical NE is characterized by diarrhea, bloody feces, intestinal ulcer erosion, peracute course, and high mortality [5]. Whereas the flock suffering from SNE presents no overt clinical signs and low mortality [4], in most cases, even only an overall reduction in the growth performance of broilers is observed [6]. As a result, SNE is difficult to diagnose and control timely, leading to more widespread infections and greater economic losses than acute clinical NE [6]. In 2015, a study reported that 27.45% of the drug-free broiler flocks suffered from acute clinical NE, and 49.02% suffered from SNE in eight commercial farms from Canada [1]. In addition, previous studies [7, 8] have demonstrated that SNE usually results in pathological changes of intestinal structure, damages of intestinal barrier function, activation of intestinal inflammatory pathways, disorders of intestinal microflora, poor digestion and absorption, and depressing growth performance of broilers. Therefore, modulation in intestinal health may be a potential strategy to prevent SNE in broiler.

With banning of antibiotics, various strategies have been used against SNE in broiler; apart from organic acids [9], polysaccharides [10], vaccines [11], prebiotics [8] and probiotics [7], extracts from natural plants have been demonstrated to be effective for its protection on broiler health [12]. Ellagic acid (EA) is a chromene-dione derivative (2,3,7,8-tetrahydroxy-chromeno [5,4,3-cde]chromene-5,10-dione; C_14_H_6_O_8_) extracted from various fruits, nuts, vegetables and herbs [13]. EA possesses numerous pharmacological functions, including anti-oxidation [14], anti-inflammation [15, 16], anti-cancer [13] and anti-metabolic syndrome [17]. In rats, EA exerted anti-inflammatory and antioxidant functions against streptozotocin -induced diabetic nephropathy via reducing the activation of NF-κB and increasing the nuclear translocation of Nrf2 to up-regulate the activities of GSH, γ-GCL and SOD [18]. Meanwhile, the alleviating effects of EA on inflammatory mediators including TNF-α, IL-1β, IL-6, IL-8 and iNOS through TLRs, NF-κB or STAT signaling pathways have also been widely reported in mice or rats [15, 16, 19]. In addition, EA can alter intestinal microbiota composition and be transformed to urolithins by some microorganism, possessing potential protection against oxidative stresses and inflammatory diseases in gastrointestinal tract of animal [20]. Ellagitannins (ETs), which can be hydrolyzed to EA in digestive tract, show a prebiotic effect on promoting the growth of *Lactobacillus* and *Bifidobacterium* [21]. In human, pomegranate ETs can increase the counts of *Akkermansia mucinifila* [22], improving metabolic functions and immune responses of the host. However, no study has investigated the protective influences and mechanisms of EA against intestinal diseases (especially SNE) in poultry to date. Therefore, this study was undertaken to explore the preventing effects and mechanisms of EA on growth performance, immune response, intestinal barrier function, antioxidant capacity, and intestinal microflora of broilers suffered with SNE induced by *C. perfringens*.

## Materials and methods

### Experimental animals and treatments

A total of 240 1-day-old male Arbor Acres broilers with an average weight at 40.4 g (SD 1.57) were purchased from Beijing Arbor Acres Poultry Breeding Co., Ltd. (Beijing, China). Upon arrival, birds were weighed and randomly assigned to four groups. Each group had six replicates with ten birds per replicate. Each replicate was reared in a cage (1.0 m × 1.0 m × 0.6 m, length × width × height) with a raised wire-netted floor. A 2 × 2 factorial design was used to investigate the effects of dietary EA level (0 or 500 mg/kg), *C. perfringens* challenge (challenged or unchallenged) and their interactions on broiler. The treatments were as follows: (1) control group (Control, basal diet); (2) *C. perfringens* challenge group (CP, basal diet + *C. perfringens* challenge); (3) ellagic acid and *C. perfringens* challenge group (EAXCP, basal diet extra 500 mg/kg ellagic acid + *C. perfringens* challenge); (4) ellagic acid group (EA, basal diet extra 500 mg/kg ellagic acid). Ellagic acid (99%, extracted from pomegranate peel) was purchased from Shaanxi Pioneer Biotech Co., Ltd. (Shanxi, China).

Corn-soybean meal basal diets were formulated according to the nutrient requirements for broilers as recommended by the National Research Council (NRC, 1994) [23]. The composition and nutrient levels of the basal diets are presented in Additional file 1: Table S1. All diets were crumbled, and neither antibiotics nor antibacterial supplements were added. To avoid cross-contamination, the unchallenged birds and *C. perfringens*-challenged birds were reared in two separate parts in a light and climate controlled room at a 23-h light/1-h dark cycle, and provided with feed and water ad libitum. Room temperature was maintained at 33 °C during the first 5 d and then gradually decreased by 5 °C weekly until 23 ± 1 °C. In addition, birds were vaccinated against Newcastle disease virus and infectious bronchitis virus vaccines on d 7 and against bursa disease virus via drinking water on d 12 and 26 according to the routine immunization program.

### *Clostridium perfringens* challenge

Avian *C. perfringens* type A field strain (CVCC2030) was obtained from State Key Laboratory of Animal Nutrition (Beijing, China). *C. perfringens* culture and challenge were performed on the basis of the previous reports [24–26] with some modifications. Briefly, *C. perfringens* was anaerobically cultured in cooked meat medium with dried meat particles (CM605, CM607; Beijing Land Bridge Technology Co., Ltd., Beijing, China) for 24 h at 37 °C, then aseptically transferred into thioglycolate broth (70157, Millipore, Shanghai, China) and incubated anaerobically for 18 h at 37 °C. Birds from CP and EAXCP groups were challenged with 1.0 mL of actively growing culture of *C. perfringens* at 2 ~ 3 × 10^8^ CFU/mL by oral gavage each day from d 16 to d 20, while those from Control and EA groups received an equal volume of thioglycolate broth.

### Growth performance

On d 21 and d 42, the birds were feed-deprived for 8 h, and then the feed intake and body weight (BW) of the birds in each replicate were measured. The average daily feed intake (ADFI), average daily gain (ADG) and feed conversion ratios (FCR, feed intake/BW gain) of the birds were calculated for d 1–21, 22–42 and 1–42, respectively.

### Sample collection

At d 42, one bird per replicate was randomly selected for blood samples collection by wing vein puncture. Serum was separated by centrifugation at 3000 r/min for 10 min at 4 °C. After the birds were euthanized by jugular exsanguination, approximately 1 cm jejunal samples between Meckel’s diverticulum and the proximal of jejunum were collected, and snap-frozen in liquid nitrogen; approximately 2 cm jejunal samples in length midway between the endpoint of the duodenal loop and Meckel’s diverticulum were collected, flushed and fixed with 10% neutral buffered formalin solution for morphological analysis [26]. Jejunal mucosa were scraped from the posterior part half of jejunum. Cecal content samples were aseptically collected and snap-frozen in liquid nitrogen. Serum, cecal content samples and all tissues were stored at − 80 °C until analysis.

### Plasma D-xylose concentration

Plasma D-xylose concentration was measured as previously described method [24]. Briefly, at d 42, another one feed-deprived bird per replicate was randomly selected, weighed, and administered D-xylose (X1500; Sigma-Aldrich, Shanghai, China) solution at a dose of 0.1 g/kg body weight by oral gavage. After 1 h, blood samples were collected into heparinized vacuum tubes by wing vein puncture. Plasma was separated by centrifugation at 3000 r/min for 10 min at 4 °C and stored at − 80 °C. D-xylose concentration in plasma was determined using the D-xylose assay kit (A035; Nanjing Jiancheng Bioengineering Institute, Nanjing, Jiangsu, China) according to the manufacturer’s instructions.

### Serum biochemical assay

The concentrations of endotoxin lipopolysaccharide (LPS), procalcitonin (PCT), C-reactive protein (CRP) and interleukin 6 (IL-6) in serum were determined using chicken ELISA kits (YM-3864, YM-S0818, YM-3783; Shanghai YuanMu Biological Technology Co., Ltd., Shanghai, China. H007; Nanjing Jiancheng Bioengineering Institute, Nanjing, Jiangsu, China); the activities of myeloperoxidase (MPO) and diamine oxidase (DAO) in serum were determined using commercially available assay kit (A088, A044; Nanjing Jiancheng Bioengineering Institute, Nanjing, Jiangsu, China) according to the manufacturer’s instructions, respectively.

### Intestinal morphology

Fixed jejunal tissues were embedded in paraffin, then tissue rings were sliced into 5-μm thickness, deparaffinized in xylene, rehydrated and mounted on glass slides [25, 26]. These sections of jejunal tissues were stained with haematoxylin and eosin (H&E). The slides were photographed on a microscope slide scanner (3D HISTECH Ltd., Budapest, Hungary, Model Pannoramic MIDI). At least nine villi per section and two sections each sample were measured to evaluate villus height (VH) and crypt depth (CD) using CaseViewer V 2.43 (3D HISTECH Ltd., Budapest, Hungary). The mean values of villus height and crypt depth were calculated and used to obtain the villus height/crypt depth (V/C) ratio.

### Intestinal mucosa enzyme activities

The activities of inducible nitric oxide synthase (iNOS), lysozyme (LZM), superoxide dismutase (SOD), and catalase (CAT), as well as the concentration of malondialdehyde (MDA) in jejunal mucosa were determined using commercially available assay kits (A014, A050, A001, A007 and A003; Nanjing Jiancheng Bioengineering Institute, Nanjing, Jiangsu, China) according to the manufacturer’s instructions, respectively.

### Intestinal immune and tight junction-related genes expression

Total RNA was extracted from jejunal tissues using Eastep® Super Total RNA Extraction Kit (15596018; Promega Bingjing Biotech Co., Ltd., Beijing, China) according to the manufacturer’s instructions. The concentration and purity of total RNA were determined on an Ultra-micro spectrophotometer (Implen GmbH, Munich, Germany, NanoPhotometer® N60). Total RNA from each sample was reverse-transcribed into complementary DNA using a TRUEscript RT Kit (+gDNA eraser) (PC5402; Aidlab Biotechnologies CO., Ltd., Beijing, China). Two-step quantitative real-time PCR was performed with a Sybr Green qPCR Mix (PC3302; Aidlab Biotechnologies CO., Ltd., Beijing, China) on a Real-Time PCR Detection Systems (Bio-Rad, Hercules, California, USA, CFX Connect™) according to the manufacturer’s instructions. Oligonucleotide primers of inflammatory mediator genes (*TNF-α*, *IL-1β*, *IL-8*, *IFN-γ*, *TGF-β* and *iNOS*), inflammation-related signaling pathway genes (*TLR-2*, *TLR-4*, *MyD88*, *NF-κB*, *JAK1*, *JAK2*, *JAK3*, *STAT1* and *STAT6*), and tight junction-related genes (*ZO-1*, occludin and claudin-2) for chicken were designed based on databases of National Center for Biotechnology Information (U.S. National Library of Medicine, Bethesda, Maryland, USA) using Oligo V 7.0 (Molecular Biology Insights, Inc., Colorado Springs, Colorado, USA) and synthesized by Sango Biotech Co., Ltd. (Shanghai, China). Table S2 lists the quantitative real-time PCR primers used in this study. The relative mRNA expression level of each target gene was calculated based on the expression of the housekeeping gene β-actin using the 2^−ΔΔCt^ method [27].

### Cecal microbiota pyrosequencing and analysis

Bacterial DNA was extracted from cecal content samples using a QIAamp DNA Stool Mini Kit (51504; Qiagen Inc., Shanghai, China) according to the manufacturer’s instructions. The concentration and purity of total DNA were determined on an Ultra-micro spectrophotometer (Implen GmbH, Munich, Germany, NanoPhotometer® N60). V4 region of bacterial 16S rRNA gene was amplified with the barcoded primer pair 515F/806R (515F: 5′-GTG CCA GCM GCC GCG GTA A-3′, 806R: 5′-GGA CTA CHV GGG TWT CTA AT-3′) using PCR, then PCR products run on a 2% agarose gel and were purified using a QIAquick Gel Extraction Kit (28706; Qiagen Inc., Shanghai, China) according to the manufacturer’s instructions. Pyrosequencing for 16S rDNA was performed on a high-throughput sequencing platform (Illumina, Shanghai, China, HiSeq® 2500 Miseq PE250).

Sequencing results were merged using FLASH V 1.2.7 (http://ccb.jhu.edu/software/FLASH/index.shtml), filtered using QIIME V 1.9.1 (http://qiime.org), and the chimera sequences were excluded based on Silva database using UCHIME V 4.1 (http://www.drive5.com/usearch/manual/uchime_algo.html) to obtain effective tags finally. The effective tags with ≥97% similarity were assigned to the same operational taxonomic units (OTUs) using uParse V 7.0.1001 (http://www.drive5.com/uparse), and the taxonomic information of each OUT was annotated based on Silva Database using Mothur V 1.35.1 (http://mothur.org). Multiple sequence alignment was conducted using MUSCLE V 3.8.31 (http://www.drive5.com/muscle) to analyse the phylogenetic relationship between different OTUs and the difference of the dominant species among different treatment groups. OTUs abundance information was normalized based on the sample with the most minimal sequences for subsequent analysis.

Venn diagram, rarefaction curve, box plot analysis, principal co-ordinates analysis (PCoA), and bacteria relative abundance were created with R software V 2.15.3 (http://www.R-project.org). Alpha diversity including ACE, Chao1, Simpson and Shannon index were calculated using QIIME V 1.9.1 (http://qiime.org). Beta diversity was calculated from bray-curtis distance using QIIME V 1.9.1 (http://qiime.org). Line discriminant analysis effect size (LEfSe) was used to determine the significance of the difference between treatments.

All of the procedures were conducted by Novogene Bioinformatics Technology Co. Ltd. (Beijing, China).

### Statistical analysis

Data was analysed using GraphPad Prism V 8.0.1 (GraphPad Software, San Diego, California, USA). As a 2 × 2 factorial arrangement, two-way ANOVA was used to determine the main effects of dietary EA level and *C. perfringens* challenge, and their interaction, Tukey’s multiple comparison was used to separate means when interactive effects significantly different or had a trend of difference [24, 26]. Results are presented as the means ± SEMs. All statements of significance were based on *P* < 0.05, and *P* value between 0.05 and 0.10 was classified as a tendency [26].

## Results

### Growth performance

The growth performance of broilers on BW, ADG, ADFI and FCR was shown in Table 1. In present study, *C. perfringens* challenge decreased BW and ADG of broilers during d 1–21, 22–42 and 1–42 (*P* < 0.05), while increased FCR during d 22–42 and 1–42 (*P* < 0.05). The dietary supplement of EA heightened ADG of broilers during d 22–42 (*P* < 0.05) and lowered FCR during d 22–42 and 1–42 (*P* < 0.05). There was no interacting effect between dietary EA levels and *C. perfringens* challenge on the growth performance of broilers. In summary, *C. perfringens* challenge resulted in a decline on the growth performance of broilers; nevertheless EA alleviated this adverse effect and improved the growth performance of broilers.

**Table 1:** Effect of *C. perfringens* challenge and dietary EA levels on the growth performance of broilers

Dietary EA levels	0 mg/kg		500 mg/kg			*P*-values		
*C. perfringens* challenge	–	+	–	+	SEM	*C. perfringens* challenge	Dietary EA level	Interaction
**d 1**–**21**								
BW, g	563.10	533.18	555.95	535.05	6.21	0.0466*	0.8277	0.7106
ADG, g	24.89	23.47	24.55	23.56	0.29	0.0470*	0.8284	0.7111
ADFI, g	39.73	40.34	38.82	38.10	0.65	0.9669	0.2517	0.6261
FCR, g/g	1.60	1.72	1.58	1.63	0.03	0.1962	0.4186	0.5772
**d 22**–**42**								
BW, g	1695.93	1589.26	1758.52	1662.96	24.00	0.0313*	0.1342	0.9000
ADG, g	53.94	50.29	57.27	53.71	0.91	0.0367*	0.0493*	0.9756
ADFI, g	102.16	103.95	103.69	100.17	1.08	0.7002	0.6155	0.2426
FCR, g/g	1.89	2.07	1.82	1.87	0.03	0.0327*	0.0146*	0.2526
**d 1**–**42**								
BW, g	1695.93	1589.26	1758.52	1662.96	24.00	0.0313*	0.1342	0.9000
ADG, g	39.42	36.88	40.91	38.63	0.57	0.0314*	0.1339	0.9003
ADFI, g	70.94	72.15	71.25	69.13	0.79	0.7797	0.4133	0.3176
FCR, g/g	1.80	1.96	1.74	1.80	0.03	0.0467*	0.0460*	0.3100

All values are expressed as the means (*n* = 6). * Significant main effect (*P* < 0.05) of *C. perfringens* challenge or dietary EA level. *BW* Body weight, *ADFI* Average daily feed intake, *ADG* Average daily gain, *FCR* Feed conversion ratios = g of feed intake / g of BW gain, g/g

### Intestinal morphology

**Fig. 1:**
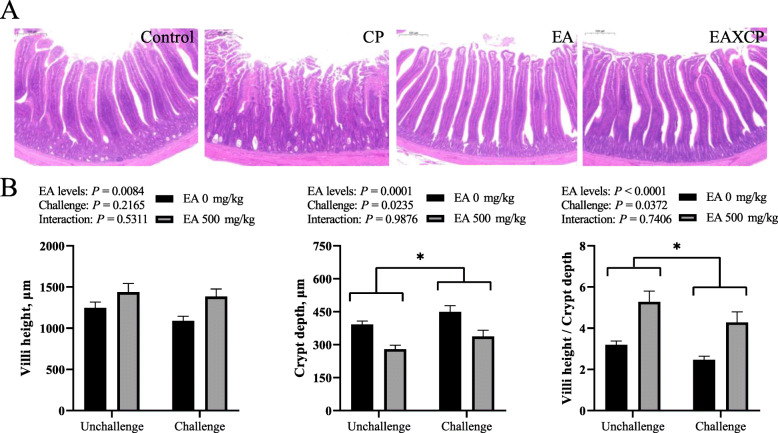
Effect of *C. perfringens* challenge and dietary EA levels on jejunal morphological parameters of broilers at d 42. (**A**) Representative photomicrographs of jejunal cross-section with HE staining. (**B**) Jejunal villi height (VH), crypt depth (CD) and villi height/crypt depth (V/C), respectively. Unchallenged, birds without *C. perfringens* infection; challenged, birds with *C. perfringens* infection. Values are means (*n* = 6) with their standard errors represented by vertical bars. * Significant main effect (*P* < 0.05) of *C. perfringens* challenge

As depicted in Fig. 1A, the jejunums of broilers in Control group demonstrated the normal appearance of intestinal villus. In contrast, the jejunums of broilers in CP group showed severe pathological changes with the disappearance of the typical villus architecture and damages of epithelial cells. The jejunal morphologies in EAXCP and EA groups exhibited no noticeable appearance change compared to those in Control group. As shown in Fig. 1B, *C. perfringens* challenge increased jejunal CD (*P* < 0.05) and decreased V/C ratio (*P* < 0.05) of broilers. On the contrary, the supplement of EA in diet exhibited a decreased effect on CD (*P* < 0.01), while an increased effect on VH and V/C ratio (*P <* 0.01) in jejunum of broilers. No interacting effect was observed between dietary EA levels and *C. perfringens* challenge on jejunal VH, CD and V/C ratio of broilers.

### Systemic inflammation

**Fig. 2:**
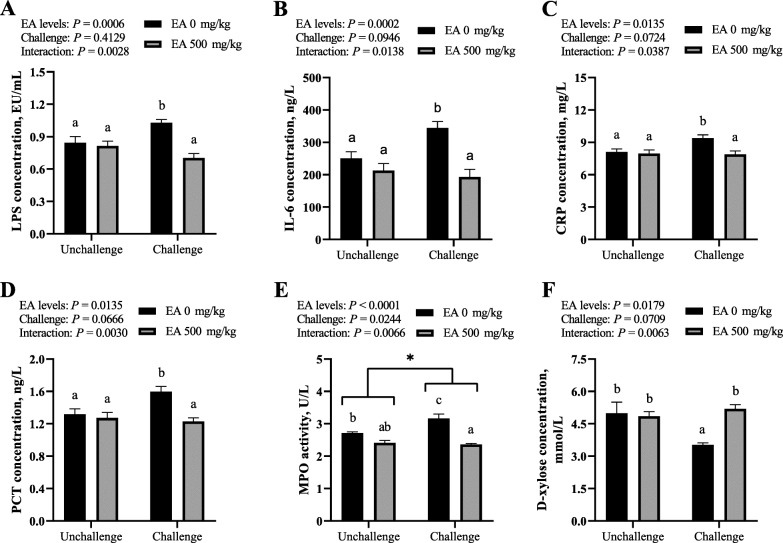
Effect of *C. perfringens* challenge and dietary EA levels on serum inflammation biomarkers and plasma D-xylose concentration of broilers at d 42. (**A**, **B**, **C**, **D** and **E**) The concentrations of endotoxin lipopolysaccharide (LPS, EU/mL), interleukin 6 (IL-6, ng/L), C-reactive protein (CRP, mg/L), procalcitonin (PCT, ng/L), and the activity of myeloperoxidase (MPO, U/L) in serum. (**F**) Plasma D-xylose concentration (mmol/L). Unchallenged, birds without *C. perfringens* infection; challenged, birds with *C. perfringens* infection. Values are means (*n* = 6) with their standard errors represented by vertical bars. ^a, b, c^ Values with unlike letters were significantly different (*P* < 0.05). * Significant main effect (*P* < 0.05) of *C. perfringens* challenge

As presented in Fig. 2 A, B, C, D and E, the systemic inflammatory response intensity was evaluated by measuring the concentrations of inflammation biomarkers LPS, IL-6, CRP and PCT, and the activity of MPO in serum of broilers. The infection of *C. perfringens* caused a heightened tendency on the concentrations of LPS, IL-6, CRP and PCT (0.05 < *P* < 0.10), and an increase on the activity of MPO (*P* < 0.05) in serum of broilers. The addition of EA in diet reduced the concentrations of CRP and PCT (*P* < 0.05), furthermore, resulted in an extreme decrease on the concentrations of LPS and IL-6 (*P* < 0.01), as well as the activity of MPO (*P* < 0.01) in serum of broilers. There was an interacting effect between dietary EA levels and *C. perfringens* challenge on the concentrations of LPS, PCT, IL-6 and CRP (*P* < 0.05), and the activity of MPO (*P* < 0.01) in serum of broilers. Multiple comparisons indicated that the concentrations of LPS, IL-6, CRP and PCT, and the activity of MPO in the serum of broilers from CP group were higher (*P* < 0.05) compared with those from the other three groups. Moreover, the serum of birds in EAXCP group possessed lower MPO activity (*P* < 0.05) than that in Control group.

### Intestinal permeability

As shown in Fig. 2F, intestinal permeability was assessed by determining the concentration of D-xylose in plasma of broilers. The concentration of plasma D-xylose emerged a decreased tendency (0.05 < *P* < 0.10) in birds with *C. perfringens* infection. On the contrary, the supplement of EA in diet enhanced its concentration (*P* < 0.05). Meanwhile, an extreme interaction (*P* < 0.01) was found between dietary EA levels and *C. perfringens* challenge on the concentration of plasma D-xylose in broilers. Furthermore, birds in CP group displayed a decreased concentration of plasma D-xylose (*P* < 0.05) relative to those in the other three groups.

### Intestinal mucosa integrity and barrier-related enzyme activities

**Fig. 3:**
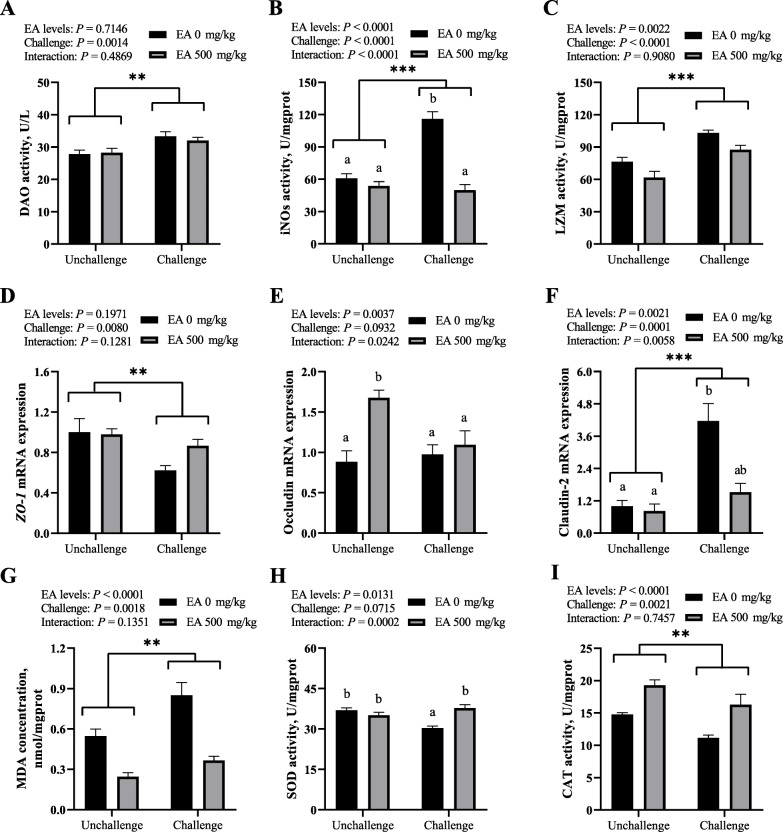
Effect of *C. perfringens* challenge and dietary EA levels on intestinal barrier-related biomarkers of broilers at d 42. (**A**) Diamine oxidase activity (DAO, U/L) in serum. (**B** and **C**) The activities of inducible nitric oxide synthase (iNOS, U/mgprot) and lysozyme (LZM, U/mgprot) in jejunal mucosa. (**D**, **E** and **F**) Relative mRNA expressions of zonula occludens 1 *(ZO-1),* occludin and claudin-2 in jejunal mucosa. (**G**, **H** and **I**) The concentration of malondialdehyde (MDA, nmol/mgprot), and the activities of superoxide dismutase (SOD, U/mgprot) and catalase (CAT, U/mgprot) in jejunal mucosa. Unchallenged, birds without *C. perfringens* infection; challenged, birds with *C. perfringens* infection. Values are means (*n* = 6) with their standard errors represented by vertical bars. ^a, b^ Values with unlike letters were significantly different (*P* < 0.05). *^,^ **^,^ *** Significant main effect (*P* < 0.05, *P* < 0.01, *P* < 0.001) of *C. perfringens* challenge

Serum DAO activity was measured to reflect the intestinal mucosa integrity of broilers, and the activities of iNOS and LZM in mucosa were used to evaluate jejunal barrier function. As described in Fig. 3A, B and C, the infection of *C. perfringens* increased the activities of DAO in serum, as well as iNOS and LZM (*P* < 0.01) in jejunal mucosa of broilers, however, dietary EA supplement decreased the activities of iNOS and LZM (*P* < 0.01). Furthermore, an interacting effect between dietary EA levels and *C. perfringens* challenge was observed on iNOS activity (*P* < 0.01). As the results of multiple comparisons, birds in CP group showed higher iNOS activity in jejunal mucosa than those in the other three groups (*P* < 0.05).

### Tight junction-related gene expression in jejunal mucosa

Figure 3D, E and F respectively exhibited the relative mRNA expressions of tight junction-related gene *ZO-1*, occludin and claudin-2 in jejunal mucosa of broilers. *C. perfringens* challenge elevated the mRNA expression of claudin-2 (*P* < 0.01) in jejunal mucosa of broilers, yet down-regulated the mRNA expression of *ZO-1* (*P* < 0.01), while caused a reduced tendency on the mRNA abundance of occludin (0.05 < *P* < 0.10). The dietary EA supplement increased the mRNA expression of occludin (*P* < 0.01) and lowered mRNA expression of claudin-2 (*P* < 0.01) in jejunal mucosa of broilers. In addition, dietary EA levels and *C. perfringens* challenge exerted an interacting effect on the relative mRNA expressions of occludin (*P* < 0.05) and claudin-2 (*P* < 0.01) in jejunal mucosa. The mRNA abundance of claudin-2 in jejunal mucosa was increased (*P* < 0.05) in CP group in contrast to Control group. Meanwhile, the birds only fed EA diet possessed higher mRNA expression of occludin (*P* < 0.05) in jejunal mucosa than those in the other three groups.

### Intestinal antioxidant capability

As shown in Fig. 3G, H and I, we determined the concentration of the lipid peroxidation product MDA and the activities of antioxidant enzyme SOD and CAT in jejunal mucosa to assess the degree of oxidative damages in broilers. The infection of *C. perfringens* increased the concentration of MDA (*P* < 0.01), decreased the activity of CAT (*P* < 0.01), and had a downward tendency on the activity of SOD (0.05 < *P* < 0.10) in jejunal mucosa of broilers, whereas adding EA to diet declined the concentration of MDA (*P* < 0.01), while heightened the activities of SOD and CAT (*P* < 0.01). An extreme interaction effect on the activity of SOD (*P* < 0.01) in jejunal mucosa was observed between dietary EA levels and *C. perfringens* challenge. Furthermore, multiple comparisons indicated that birds in CP group showed a decrease on the activity of SOD (*P* < 0.05) in jejunum compared with those in the other three groups.

### Gene expression of intestinal inflammation-related cytokine and pathway

**Fig. 4:**
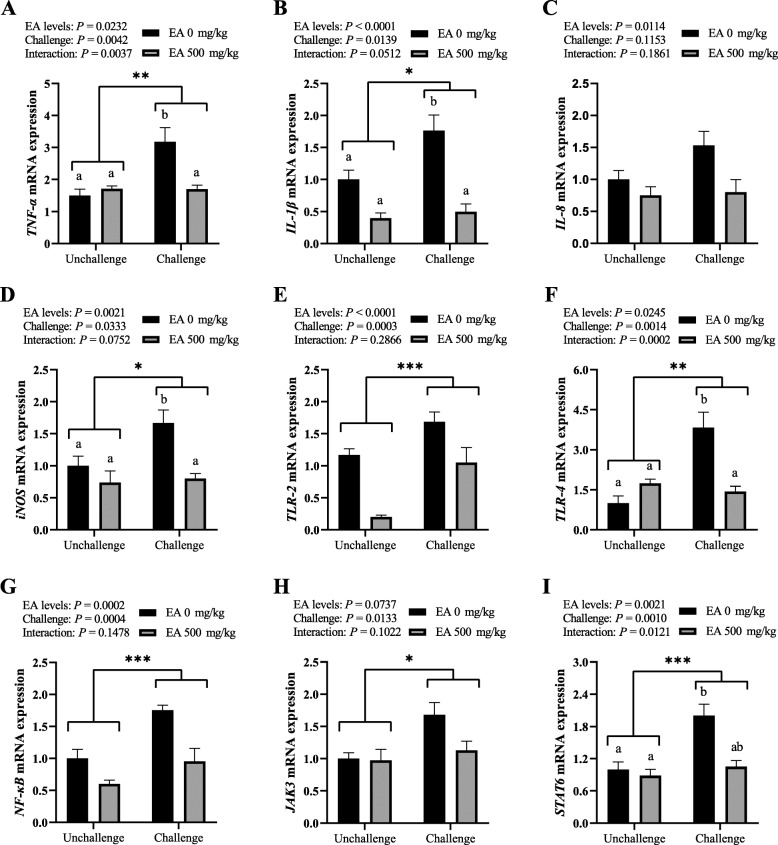
Effect of *C. perfringens* challenge and dietary EA levels on relative mRNA expression of inflammation-related pathway and cytokine genes in jejunal mucosa of broilers at d 42. (**A**, **B**, **C** and **D**) The relative mRNA expressions of tumor necrosis factor alpha (*TNF-α*), interleukin 1 beta (*IL-1β*), interleukin 8 (*IL-8*) and inducible nitric oxide synthase (*iNOS*). (**E**, **F** and **G**) The relative mRNA expressions of toll-like receptor 2 (*TLR-2*), toll-like receptor 4 (*TLR-4*) and nuclear factor kappa B (*NF-κB*). (**H** and **I**) The relative mRNA expression of janus kinase 3 (*JAK3*) and signal transducers and activators of transcription 6 (*STAT6*). Unchallenged, birds without *C. perfringens* infection; challenged, birds with *C. perfringens* infection. Values are means (*n* = 6) with their standard errors represented by vertical bars. ^a, b^ Values with unlike letters were significantly different (*P* < 0.05). *^,^ **^,^ *** Significant main effect (*P* < 0.05, *P* < 0.01, *P* < 0.001) of *C. perfringens* challenge

The relative mRNA expressions of various inflammatory mediator genes *TNF-α*, *IL-1β*, *IL-8*, *iNOS*, *TGF-β* and *IFN-γ* in jejunal mucosa were exhibited in Fig. 4A, B, C and D, as well as S1A and B, respectively. *C. perfringens* challenge caused up-regulations on the mRNA expressions of *TNF-α* (*P* < 0.01), *IL-1β* and *iNOS* (*P* < 0.05) in jejunal mucosa, but the EA diets down-regulated the mRNA abundances of *IL-1β*, *iNOS* (*P* < 0.01), *TNF-α* and *IL-8* (*P* < 0.05). Between dietary EA levels and *C. perfringens* challenge, an interaction effect was presented on the mRNA expression of *TNF-α* (*P* < 0.01), as well as a tendency on *IL-1β*, *iNOS* and *TGF-β* (0.05 < *P* < 0.10). Birds in CP group exhibited higher mRNA expressions of *TNF-α*, *IL-1β* and *iNOS* (*P* < 0.05) than those in the other three groups. But no significant difference was found on the mRNA expressions of *TGF-β* and *IFN-γ* (*P* > 0.05) in jejunal mucosa of birds*.*

The relative mRNA expressions of inflammation-related signaling pathway TLR/NF-κB genes *TLR-2*, *TLR-4*, *NF-κB* and *MyD88* in jejunal mucosa were shown in Fig. 4E, F and G, as well as S1C, respectively. The infection of *C. perfringens* increased the mRNA expressions of *TLR-2*, *TLR-4* and *NF-κB* (*P* < 0.01) in jejunal mucosa, whereas the addition of EA in diets decreased the mRNA abundances of *TLR-2*, *NF-κB* (*P* < 0.01) and *TLR-4* (*P* < 0.05). Meanwhile, an interacting effect was presented on the mRNA expression of *TLR-4* (*P* < 0.01) in jejunal mucosa of broilers between dietary EA levels and *C. perfringens* challenge. The results of multiple comparisons indicated birds in CP group shown an up-regulated mRNA expression of *TLR-4* (*P* < 0.05) compared with those in the other three groups. However, no significant difference was found on the relative mRNA expression of *MyD88* (*P* > 0.05) in jejunal mucosa of birds.

Figure 4H and I, as well as S1D, E and F respectively exhibited the relative mRNA expressions of inflammation-related signaling pathway *JAK/STAT* genes *JAK3*, *STAT6*, *JAK1*, *JAK2* and *STAT1* in jejunal mucosa of broilers. *C. perfringens* challenge resulted in an up-regulation on the mRNA expressions of *JAK3* (*P* < 0.05) and *STAT6* (*P* < 0.01), but the supplement of EA in diet led to a down-regulation on the mRNA expression of *STAT6* (*P* < 0.01) and a downward trend on *JAK3* (0.05 < *P* < 0.10) in jejunal mucosa. Moreover, dietary EA levels and *C. perfringens* challenge caused an interacting effect on the mRNA abundances of *STAT6* (*P* < 0.05) and a tendentious interaction on *JAK1* (0.05 < *P* < 0.10) in jejunal mucosa. As the results of multiple comparisons, an up-regulated mRNA expression of *STAT6* (*P* < 0.05) was shown for birds in CP group compared with those in Control group. Nevertheless, no significant difference was found on the mRNA expressions of *JAK1*, *JAK2* and *STAT1* (*P* > 0.05) in jejunal mucosa of birds*.*

### Cecal microbiota

A total of 2,401,670 pairs of reads were generated after 16S rRNA sequencing of 23 cecal digesta samples (There were only five replicates in EAXCP Group, because one sample was damaged). After splicing, filtering and removing chimeras, we obtained 1,488,682 effective Tags or 64,725 ± 613 effective Tags for each sample. Based on 97% sequence similarity, Tags were clustered into 1637 OTUs, of which four groups shared 888 OTUs, and only 76, 92, 129 and 100 OTUs were exclusive in Control, CP, EA and EAXCP groups, respectively (Fig. S2A). The Good’s coverage estimators (Table S3) and the rarefaction curves (Fig. S2B) indicated that sufficient sequencing coverage was achieved.

The alpha diversity of cecal microbiota was shown in Table S4, which exhibited the supplement of EA in diet increased observed species and Shannon index (*P* < 0.05). The beta diversity analysis was illustrated via Box and PCoA plots in Fig. S2C and D, showing no difference in the microbial community structure among groups.

The most abundant (top 10) phyla and genus of cecal microbiota were presented in Fig. S3. At the phylum level, the cecal microbiota was dominated by Firmicutes (36 ~ 53%), Bacteroidota (26 ~ 35%), Verrucomicrobiota (5 ~ 12%) and Euryarchaeota (3 ~ 7%), together making up over 86% of the total sequences. *C. perfringens* challenge increased the relative abundance of Firmicutes (*P* < 0.05) and decreased the relative abundance of Desulfobacterota (*P* < 0.05). Similarly, EA increased the relative abundance of Firmicutes (*P* < 0.05) and showed a lowering trend on the relative abundance of Desulfobacterota and Campilobacterota (0.05 < *P* < 0.10). Meanwhile, dietary EA levels and *C. perfringens* challenge led an interacting effect on the relative abundance of Firmicutes (*P* < 0.05) and a trend on Elusimicrobia (0.05 < *P* < 0.10). Multiple comparisons showed that the cecal microbiota in EAXCP group possessed the higher relative abundance of Firmicutes (*P* < 0.05) than those in the other three groups. At the genus level, only the main effect of *C. perfringens* challenge showed a heightening trend on the relative abundance of *Ruminococcus torques *group (0.05 < *P* < 0.10).

**Fig. 5:**
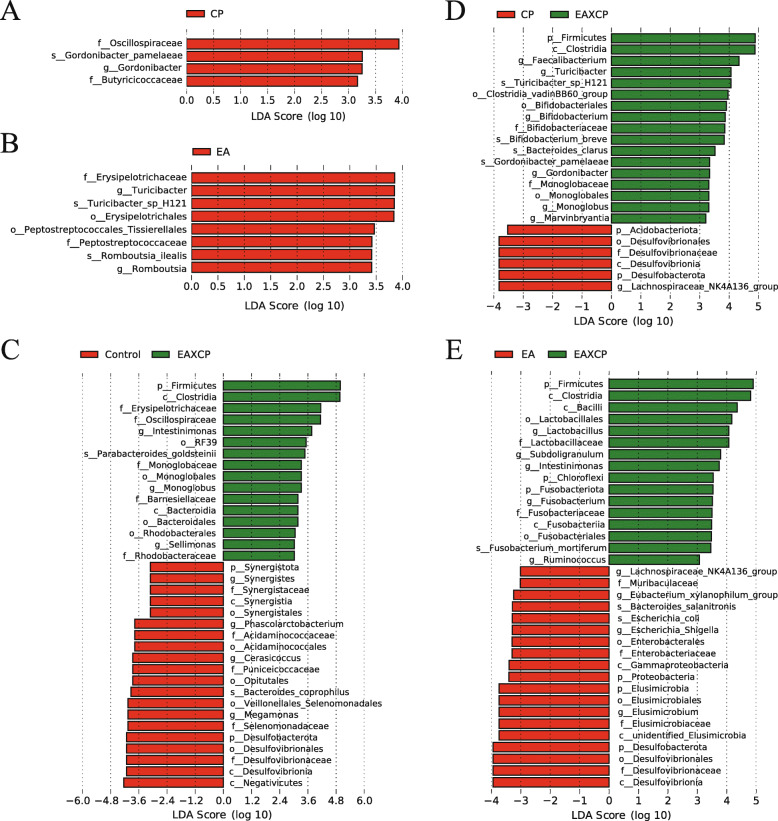
LEfSe analysis revealed the different phylotypes of cecal microbiota between groups. (**A**) Cecal microbiota between Control and CP groups. (**B**) Cecal microbiota between Control and EA groups. (**C**) Cecal microbiota between Control and EAXCP groups. (**D**) Cecal microbiota between CP and EAXCP groups. (**E**) Cecal microbiota between EA and EAXCP groups. These figures showed the bacteria of which the LDA Score is greater than the set value (the default setting was 3.0) between groups. The length of the histogram represents the size of the difference species (i.e., LDA Score), and the different colors represent the different groups

LEfSe analysis was used to determine the statistically difference between groups. Compared with cecal microbiota in Control group, Butyricicoccaceae, *Gordonibacter pamelaeae*, *Gordonibacter* and Oscillospiraceae were higher in CP group (Fig. 5A); Peptostreptococcaceae, Peptostreptococcales Tissierellales, *Romboutsia*, *Romboutsia ilealis*, Erysipelotrichales, Erysipelotrichaceae, *Turicibacter* and *Turicibacter* sp. H121 were enhanced in EA group (Fig. 5B); Rhodobacteraceae, *Sellimonas*, Rhodobacterales, Bacteroidales, Bacteroidia, Barnesiellaceae, *Monoglobus*, Monoglobales, Monoglobaceae, *Parabacteroides goldsteinii*, RF39, *Intestinimonas*, Oscillospiraceae, Erysipelotrichaceae, Clostridia and Firmicutes were more abundant, while Negativicutes, Desulfovibrionia, Desulfovibrionaceae, Desulfovibrionales, Desulfobacterota, Selenomonadaceae, *Megamonas*, Veillonellales Selenomonadales, *Bacteroides coprophilus*, Opitutales, Puniceicoccaceae, *Cerasicoccus*, Acidaminococcales, Acidaminococcaceae, *Phascolarctobacterium*, Synergistota, Synergistia, Synergistales, Synergistaceae and *Synergiste* were lower in EAXCP group (Fig. 5C). In contrast to cecal microbiota in EAXCP group, those in CP group had more abundance in *Lachnospiraceae *NK4A136 group, Desulfobacterota, Desulfovibrionia, Desulfovibrionaceae, Desulfovibrionales and Acidobacteriota, but less abundance in *Marvinbryantia*, *Monoglobus*, Monoglobales, Monoglobaceae, *Gordonibacter*, *Gordonibacter pamelaeae*, *Bacteroides clarus*, *Bifidobacterium breve*, *Bifidobacterium*, Bifidobacteriaceae, Bifidobacteriales, Clostridia vadinBB60 group, *Turicibacter *sp. H121, *Turicibacter, Faecalibacterium*, Clostridia and Firmicutes (Fig. 5D). The cecal microbiota in EA group possessed more enriched Desulfovibrionia, Desulfovibrionaceae, Desulfovibrionales, Desulfobacterota, *Elusimicrobium*, unidentified Elusimicrobia, Elusimicrobiaceae, Elusimicrobiales, Elusimicrobia, Proteobacteria, Gammaproteobacteria, Enterobacteriaceae, Enterobacterales, *Escherichia Shigella*, *Escherichia coli*, *Bacteroides salanitronis*, *Eubacterium xylanophilum *group, Muribaculaceae, *Lachnospiraceae *NK4A136 group, while less *Ruminococcus*, *Fusobacterium mortiferum*, *Fusobacterium*, Fusobacteriales, Fusobacteriia, Fusobacteriaceae, Fusobacteriota, Chloroflexi, *Intestinimonas*, *Subdoligranulum*, *Lactobacillus*, Lactobacillaceae, Lactobacillales, Bacilli, Clostridia and Firmicute (Fig. 5E) compared with those in EAXCP group.

## Discussion

**Fig. 6:**
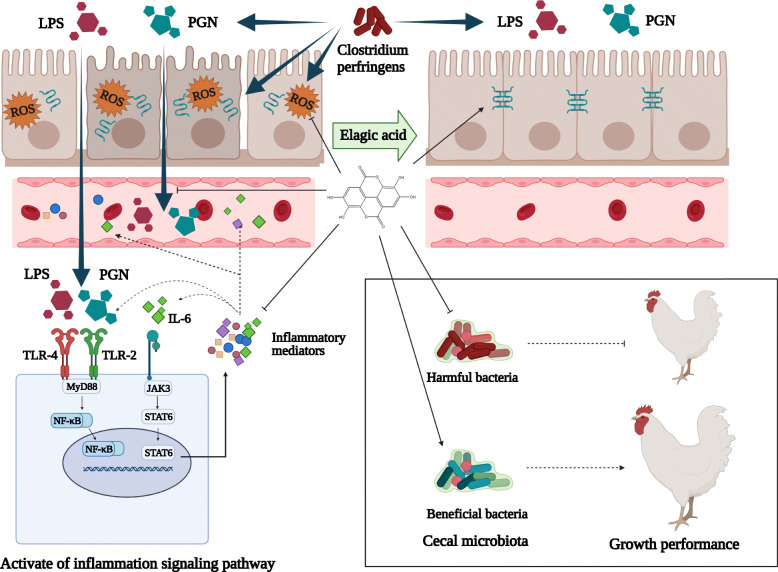
Dietary ellagic acid ameliorated *C. perfringens*-induced SNE in broilers via regulating inflammation signaling pathways TLR/NF-κB and JAK3/STAT6, as well as cecal microbiota to inhibit intestinal barrier damage. ↑ Arrows indicate the effect of stimulation; ┬ Arrows indicate the effect of suppression

In summary, this study indicated that *C. perfringens* challenge caused damages on jejunal barrier of broilers and increased the permeability of jejunal mucosa, allowing antigenic substances (LPS, etc.) to enter the blood and internal environment, which in turn triggering jejunal inflammation and oxidative stress, as well as systemic inflammation, reducing the ability of intestinal digestion and absorption, finally impairing the growth performance of broilers. However, dietary EA supplementation enhanced anti-inflammatory, antioxidant effects and intestinal barrier of jejunal mucosa, which preventing the invasion of antigenic substances, and finally improving the growth performance of broilers. Meanwhile, the supplementation of dietary EA also relieved the imbalance of cecal microbiota caused by the *C. perfringens* challenge, protecting the health of broilers (Fig. 6).

Toll-Like Receptors (TLRs) are important members of pattern recognition receptors, TLR-4 could recognize LPS which is unique to Gram-negative bacteria, and TLR-2 could recognize peptidoglycans (PGN) which is abundant in Gram-positive bacteria [28]. TLRs can trigger subsequent inflammatory responses through MyD88 dependent or independent signaling pathways that activate NF-κB and finally lead to the release of pro-inflammatory mediator including TNF-α, IL-1β, IL-6, IL-8 and iNOS [28]. In inflammatory bowel disease, LPS or cytokines (e.g. IL-6 and IFN-γ) can activate JAK/STAT signaling pathway to regulate the expression of pro-inflammatory mediators including Claudin-2 and iNOS [29]. In this study, *C. perfringens* challenge increased the mRNA expressions of TLR-4, TLR-2, NF-κB, JAK3 and STAT6, while dietary EA supplement relieved these adverse effects. Due to the deficiency of appropriate chicken-derived antibodies, we did not determine the protein levels and phosphorylation status of components in these signaling pathways. A series of studies [7, 12, 30] have reported the activation process of TLR/NF-κB or JAK/STAT signaling pathways in broilers with *C. perfringens* challenge. Similar to our results, EA was proved to possess a protective effect on concanavalin A-induced hepatitis in mice via decreasing the expressions of TLR2 and TLR4, and suppressing NF-κB signaling pathway [19]. *C. perfringens* challenge in this study has no obvious effect on the mRNA expression of *MyD88*, which might indicate TLRs activate NF-κB through MyD88 independent signaling pathways. Some studies [29, 31] have reported EA inhibited the phosphorylations of JAK1, JAK2, STAT1 and STAT3 to exert anti-inflammatory effects in keratinocytes or rats, but no study on EA regulating JAK3/STAT6 signaling pathway has been found in any animals. In human and mice, the activation of JAK3/STAT6 signaling pathway was related to the differentiation of monocytes and the enhancement of Th2 inflammatory response (the release of IL-4, IL-5 and IL-13) [32]. It means that *C. perfringens* challenge may trigger the Th2 inflammatory response related to the JAK3/STAT6 signaling pathway in jejunal mucosa of broilers, while dietary EA relieves this hazard in this pathway.

During the inflammatory response, the activation of TLR/NF-κB and JAK/STAT signaling pathways can induce the release of a variety of pro-inflammatory cytokines, which will lead to the activation of immune cells and the production of more cytokines [33]. TNF-α and IL-1β are pleiotropic pro-inflammatory cytokines, whose dysregulations are linked with a wide range of pathological conditions, such as infection, metabolic syndrome and inflammatory bowel disease [33]. IL-8 is a very potent trigger to immune cells’ migration and proliferation, which guides neutrophils to the direction of inflammation [33]. TGF-β and IFN-γ also play an important role in a variety of inflammation-related diseases. C-reactive protein promotes the inflammatory response of atrial fibrillation through the overexpression of TGF-β related to the TLR-4/NF-κB/TGF-β signaling pathway in HL-1 cells [34], while IFN-γ was reported to contribute to the hepatic inflammation in HFD-induced nonalcoholic steatohepatitis by STAT1β/TLR-2 signaling pathway in mice [35]. The expression of iNOS and the increase of NO levels can cause various inflammation-related pathophysiological conditions. The cell wall components (mainly through LPS) of bacteria can activate the JAK/STAT signaling pathway and subsequently activate NF-κB to initiate iNOS transcription [36]. In our study, *C. perfringens* challenge up-regulated the mRNA expressions of pro-inflammatory mediator genes *TNF-α*, *IL-1β* and *iNOS* in jejunal mucosa of broilers, while the EA diets down-regulated the mRNA abundances of *TNF-α*, *IL-1β*, *IL-8* and *iNOS*. A series of studies [12, 24, 30] have proved that *C. perfringens* challenge can cause an up-regulation on pro-inflammatory mediator genes including *TNF-α*, *IL-1β*, *IL-8*, *TGF-β*, *IFN-γ* and *iNOS* in the intestine of broilers. Meanwhile, the alleviating effects of EA on inflammatory mediators including TNF-α, IL-1β, IL-8 and iNOS have been widely reported in mice or rats [15, 16], which are in line with our results and further indicate that EA reduced inflammatory mediators in broilers probably through NF-κB and STAT signaling pathways. However, the *C. perfringens* challenge or dietary EA levels show no significant effect on *TGF-β* and *IFN-γ* in this study, which may be related to the type of *C. perfringens* strains and duration of the challenge.

The activation of inflammatory pathways and the release of inflammatory mediators can affect the antioxidant, barrier, and absorption functions of the jejunum, which are vital to the growth performance and health of broilers.

Oxidative stress plays an important role in NE. SOD can convert O_2_^•−^ into H_2_O_2_, then CAT transforms H_2_O_2_ into H_2_O, thus preventing the harmful effects of oxidative radicals [37]. In this study, *C. perfringens* challenge decreased the antioxidant capacity of jejunal mucosa by reducing the activities of SOD and CAT, and increasing the concentration of MDA; nevertheless, the dietary EA supplementation relieved these adverse effects and improved the health of birds. EA itself has good antioxidant capacity [14]. Moreover, in the oxidized fish oil-induced oxidative stress of mice, the supplementation of EA in diet increased the total antioxidant capacity (T-AOC) and the activities of the glutathione peroxidase (GSH-Px) and SOD, while decreased the MDA concentration in the intestine [38]. Another report demonstrated that EA exerted anti-inflammatory and antioxidant functions against streptozotocin-induced diabetic nephropathy in rats via reducing the activation of NF-κB and increasing the nuclear translocation of Nrf2 to up-regulate GSH, γ-GCL and SOD activities [18].

Tight junction proteins are vital structures of the physical barrier in jejunal mucosa, which form a seal between intestinal epithelial cells and prevent the transmission of macromolecules [7]. In the present study, *C. perfringens* challenge decreased the jejunal mRNA expressions of *ZO-1* and occludin of broilers and increased the mRNA expression of claudin-2, while the dietary supplementation of EA relieved these adverse effects. ZO-1 and occludin are barrier-forming proteins, whose reduction mean damage of tight junctions; whereas claudin-2 is a pore-forming protein, whose increase can increase the permeability of intestinal barrier [24]. As many studies reported [9, 12], the infection of *C. perfringens* can reduce the mRNA expressions of *ZO-1* and occludin in broilers through the activation of NF-κB signaling pathway. While pomegranate and pomegranate leaf, which are rich in EA, can relieve the decrease of ZO-1 and occludin caused by alcoholic liver disease or hyperlipidemia in the intestine of mice [17]. The infection of *C. perfringens* can increase the expression of claudin-2 in intestine of broilers [24], which may be explained as a result of ‘cross-talk’ caused by IL-6 among JAK/STAT, SAP/MAPK and PI3K signaling pathways [39]. Interestingly, the mRNA expression of occludin was increased in the broilers only fed the diet with EA supplementation; in another study [12], thymol and carvacrol supplementation demonstrated a similar effect on the mRNA expression of occludin in broilers challenged with *C. perfringens*.

D-xylose crosses the intestinal mucosa via a Na^+^-dependent mobile-carrier mechanism. In the case of malabsorption syndrome, the entry of D-xylose from the gut lumen to the portal vein is damaged, resulting in reduced concentration of D-xylose in blood [24]. DAO is an intracellular enzyme in the small intestinal epithelia, which would be released into the peripheral circulation as a result of intestinal villi damage, so the level of serum DAO could reflect the severity of intestinal mucosal injury [40]. In this study, the decrease of plasma D-xylose concentration indicated that *C. perfringens* challenge had impaired the intestinal absorption function, while the increase of DAO activity in serum might be related to the impaired intestinal epithelium. The supplement of dietary EA alleviated the decrease of plasma D-xylose concentration induced by *C. perfringens*, but had no effect on DAO activity in serum. Similar to our results, the arginine additive alleviated an increase on plasma D-xylose concentration caused by the *C. perfringens* challenge [24]. LZM can cleave peptidoglycan of the cell wall in Gram-positive bacteria, resulting in the loss of cellular membrane integrity and cell death [26]. In our results, *C. perfringens* infection increased the activities of iNOS and LZM in jejunal mucosa, while the supplement of EA in diet relieved these adverse effects. LZM was up-regulated in the gastrointestinal tract of patients suffering from chronic inflammation, which was related to the LZM-mediated processing of luminal bacteria in the colon that triggered the pro-inflammatory response [41]. These up-regulations of iNOS and LZM in present study further explained the mechanism of chronic inflammation caused by SNE.

Damages of the intestinal barrier and absorption function were intuitively reflected in the microstructure of intestine. *C. perfringens* challenge seriously destroyed the villi structure of jejunum and reduced the absorption surface on nutrients, which is in line with the results reported previously [12, 25]. On the contrary, the dietary EA supplementation alleviated the jejunal lesions in *C. perfringens*-challenged birds, maintaining the good condition of enterocytes and efficient absorption of nutrients. In the mice model [38], EA effectively alleviated the intestinal damage caused by oxidized fish oil via increasing the VH and V/C ratio, and relieving the injury of mucous epithelium. It has been reported that thymol and carvacrol alleviated the ileal lesion via improving V/C ratio in broilers with *C. perfringens* infection [12]. Furthermore, its antioxidant and anti-inflammatory functions may explain the mechanism how EA prevent *C. perfringens* from damaging intestinal villus-crypt architecture.

Intestinal NE lesions and mucosal atrophy greatly compromises epithelial permeability and mucosal barrier function, which may result in adverse effects on internal environment homeostasis and production performance of broilers, therefore, some serum inflammation biomarkers were used to evaluate the systemic inflammatory response intensity of broilers. LPS is an endotoxin produced by Gram-negative bacteria, its increase in blood reflected the bacteria translocation to liver, spleen and blood [42]. IL-6 is an important cytokine of inflammatory bowel diseases, which can activate the JAK/STAT signaling pathway and promote the release of various inflammatory factors [29]. CRP is synthesized in liver, mainly in response to IL-6, and can be combined with the pathogen LPS to activate the classical complement pathway [43]. PCT is a diagnostic marker of bacterial infection, which is produced by LPS, TNF-α and IL-6 acting on neuroendocrine cells or special cells in the liver and spleen [44]. MPO is a sign of neutrophil aggregation and inflammation, its activity is a marker of neutrophil infiltration into the intestine [44]. *C. perfringens* infection increased the concentrations or activities of LPS, IL-6, CRP, PCT and MPO, causing a systemic inflammatory response to broilers, while the supplement of EA in diet relieved these adverse effects. In line with our results, dietary *Lactobacillus acidophilus* supplementation significantly decreased the serum LPS content in broilers with *C. perfringens* challenge [29], while EA treatment can decrease the mRNA expressions of *TNF-α* and *IL-6* in the liver and intestine of oxidative stress mice [38]. In broilers suffering from *C. perfringens*-induced NE, probiotic powder containing *Lactobacillus plantarum* decreased the MPO activity in the mucosa of ileum [37]. Overall, the present results reflected that EA alleviated the systemic inflammatory response caused by *C. perfringens* challenge, possibly via protecting the integrity of intestinal mucosa and reducing the expression of inflammatory mediators.

On the other hand, intestinal microbiota is involved in intestinal nutrition, defense and immunity. The high diversity of intestinal microbiota is beneficial to maintaining the stability of the intestinal microenvironment and defending against the invasion of pathogenic microorganisms [45]. In this study, only the dietary EA supplement increased the alpha diversity, including observed species and Shannon index, which might mean an improvement in intestinal health. However, the beta diversity analysis showed the microbial community structure had no difference among groups, which might be related to sample resource (e.g. jejunum, colon or cecum) and the time of sample collection (e.g. at d 35 or 42). In broilers challenged with *C. perfringens* and *Eimeria* [46], the effects of dietary lauric acid supplement or the challenge on microbiota in the jejunum were distinct from microbiota in the cecum, as a result, the change of microbiota was more significant in jejunum, but the taxa abundance or diversity had no difference in cecum, which was line with our results. In terms of microbial abundance, both dietary EA supplement and *C. perfringens* challenge increased the relative abundance of Firmicutes and decreased the relative abundance of Desulfobacterota. The increase in relative abundance of Firmicutes was believed to improve the utilization of energy in the diet, and the ratio of Firmicutes to Bacteroides was often positively associated with weight gain [25]. The effect of *C. perfringens* challenge on cecal microbiota might be explained that the longer time interval between *C. perfringens* challenge and sample collection has activated the immune function of broilers and renewed the balance of cecal microbiota, especially in the EAXCP group, the *C. perfringens* challenge has played an immune-stimulating effect like vaccines with the presence of EA. In rats with stress-induced depressive-like behavior [47], fecal microbiota transplantation ameliorates gut microbiota imbalance and intestinal barrier damage through increasing the relative abundance of Firmicutes and decreasing Desulfobacterota and Bacteroidetes at phylum levels; this treatment also reduced the loss of villi and epithelial cells, suppressed the inflammatory cell infiltration, and increased the expression of ZO-1 and occludin in the ileum, which results were amazingly similar to ours. *Campylobacter* was believed to be closely related to the zoonotic campylobacter disease [48], so the EA-induced decrease in the relative abundance of Campilobacterota might exert a protective effect on the health of broilers. At the genus level, only the main effect of *C. perfringens* challenge showed a heightening trend on the relative abundance of *Ruminococcus torques *group. The increase of *Ruminococcus torques *group was reported in irritable bowel syndrome, which was associated with severity of bowel symptoms [49]. Another study believed that *Ruminococcus torques *group seemed to be especially involved in controlling paracellular permeability [50].

LEfSe analysis revealed the different phylotypes of cecal microbiota between groups. Compared with cecal microbiota in Control group, the increased abundance of Oscillospiraceae in CP group was thought to be linked to intestinal inflammation [51]. Butyricicoccaceae was an important butyrate producer [51], which might be beneficial to the recovery of the intestines. *Gordonibacter pamelaeae* has been reported to have the function of transforming EA into urolithin [20], its high abundance was observed in the EAXCP group. The increase on the abundances of *Turicibacter *sp. H121 was observed in cecal microbiota of birds fed with EA, but its effect mechanism was unclear. Compared with cecal microbiota in Control group, an increase on the abundance of *Romboutsia ilealis* in EA group was found. *Romboutsia ilealis* is a beneficial bacterium in intestine, whose decrease was considered to be harmful in zebrafish infected with *Streptococcus agalactiae* [52]. The cecal microbiota in EAXCP group was quite different from the other three groups. The increased abundance of *Sellimonas* has been reported as a potential biomarker of homeostasis gut recovery after dysbiosis events [53], Bacteroidales was thought to be involved in the synthesis of fatty acids and was beneficial to the health of the host [54], Erysipelotrichaceae was highly abundant in good FCR broilers [55], and mice fed with normal diet possessed more abundant of Monoglobaceae and RF39 than those fed with high fat diet [56]. Rhodobacteraceae is widely reported in aquatic animals or marine environments and has no adverse effects on host health. The abundance of *Synergistes* was reported to be negatively correlated with the levels of IL-1β, IL-6 and TNF-α in serum of piglets [57], but *Phascolarctobacterium* predominated among the *Clostridia* in low FCR birds [58]. Dietary supplementation with medium-chain a-monoglycerides can decrease the abundance of *Cerasicoccus,* and improve productive performance and egg quality in aged hens [58]. Compared with birds in CP group, those in EAXCP group had higher abundance of *Faecalibacterium*, which was enriched in chickens with the higher BW [59]. It was speculated that Clostridiales vadinBB60 group might also be beneficial bacteria in intestinal tract of broilers [7]. *Bifidobacterium breve* has been verified to be probiotic [60]. Comparing with birds in EA group, those in EAXCP group had a higher abundance of *Subdoligranulum*, which was negatively correlated with CRP and IL-6 in human [61]. Some strains of *Fusobacterium mortiferum* isolated from poultry caeca can produce bacteriocin-like substances to inhibit *Salmonella enteritidis* [62]. Moreover, birds in EA group had higher abundance of Muribaculaceae, which negatively correlated with inflammatory markers in high fat-high sucrose diet-induced insulin resistant mice [63]. *Eubacterium xylanophilum *group was thought to be lactic acid- and SCFA-producing bacteria, which could enhance intestinal homeostasis and ameliorate weaning stress in piglets [64]. *Escherichia coli* showed higher levels in broilers with smaller BW [59]. *Elusimicrobium* was thought to be beneficial bacteria, whose increase can protect the intestinal barrier in rats [65]. Overall, *C. perfringens* challenge caused an adverse effect on the cecal microbiota of broilers, dietary EA supplementation led to a small beneficial effect, while the simultaneous effect of dietary EA and challenge seemed to stimulate the immune function of broilers and improved the balance of cecal microbiota. Furthermore, the cecal microbiota of the EAXCP group seemed to be very different from other groups, which might explain the significant interaction between dietary EA level and *C. perfringens* challenge in our results.

Finally, growth performance is the most comprehensive indicator of commercial broiler quality. SNE induced by *C. perfringens* usually reduces the performance of broilers without serious clinical symptoms and high mortality [4, 6]. Previous studies [26, 42] reported that *C. perfringens* challenge reduced body weight gain and feed intake, while heighten FCR of broilers, which were similar to our results. The supplementation of plant extracts, including tannin and polyphenol compounds, have been proved to be effective against NE [46, 66], but the effect of EA on the growth performance of broilers has not been reported. In this study, the addition of EA in diet increased ADG and decreased FCR of broilers. In laying quails, EA improved the feed conversion and egg quality [67]. In addition, pomegranate extract was reported to have a positive effect on the growth and slaughter performances of broilers [68]. These improvements provided by EA may be explained by improving the intestinal barrier function and microbiota structure, thereby indirectly increasing the performance of broilers.

## Conclusion

In summary, this study found that dietary ellagic acid ameliorated *C. perfringens*-induced SNE in broilers via regulating jejunal inflammation signaling pathways TLR/NF-κB and JAK3/STAT6, relieving jejunal oxidative stress and balancing cecal microbiota to inhibit intestinal barrier damage, prevent systemic inflammatory response and improve nutrient absorption, finally enhance growth performance of broilers.

## Supplementary Information

**Additional file 1: Table S1.** Composition and nutrient levels of the basal diets. **Table S2.** Primers used for quantitative real-time PCR. **Table S3.** Good’s coverage estimators of *C. perfringens* challenge and dietary EA levels treatments. **Table S4.** Effect of *C. perfringens* challenge and dietary EA levels on alpha diversity of cecal microbiota. **Fig. S1.** Effect of *C. perfringens* challenge and dietary EA levels on relative mRNA expression of jejunal inflammation-related pathway and cytokine genes in jejunal mucosa of broilers at d 42. (**A** and **B**) The relative mRNA expressions of transforming growth factor-β (*TGF-β*) and interferon γ (*IFN-γ*). (**C**) The relative mRNA expression of myeloiddifferentiationfactor88 (*MyD88*). (**D**, **E** and **F)** The relative mRNA expression of Janus kinase 1 (*JAK1*), Janus kinase 2 (*JAK2*), and signal transducers and activators of transcription 1 (*STAT1*). Unchallenged, birds without *C. perfringens* infection; challenged, birds with *C. perfringens* infection. Values are means (*n* = 6) with their standard errors represented by vertical bars. **Fig. S2.** The quality of sequencing data and beta diversity of cecal microbiota. (**A**) Venn diagram of the OTUs. (**B**) The rarefaction curve analysis of the microbial species. (**C** and **D**) Box plot and principal co-ordinates analysis (PCoA) plot of beta diversity. All values are expressed as the means (*n* = 5 in EAXCP group, *n* = 6 in the other three groups). **Fig.S3.** The most abundant (top 10) phyla and genus of cecal microbiota. (**A**) The most abundant (top 10) phyla of cecal microbiota. (**B**) The most abundant (top 10) genus of cecal microbiota. (**C**, **D**, **E**, **F** and **G**) The relative abundance of Firmicutes, Desulfobacterota, Campilobacterota, Elusimicrobia and *Ruminococcus torques *group.

## Data Availability

The datasets used and/or analyzed during the current study are available from the corresponding author on reasonable request.

## Abbreviations

NE: Necrotic enteritis
SNE: Subclinical necrotic enteritis
EA: Ellagic acid
CP: Crude protein
ADFI: Average daily feed intake
BW: Body weight
ADG: Average daily gain
FCR: Feed conversion ratios
H&E: Haematoxylin and eosin
CD: Crypt depth
VH: Villi height
MPO: Myeloperoxidase
LPS: Lipopolysaccharide
DAO: Diamine oxidase
IL-6: Interleukin 6
CRP: C-reactive protein
PCT: Procalcitonin
iNOS: Inducible nitric oxide synthase
LZM: Lysozyme
ZO-1: Zonula occludens 1
MDA: Malondialdehyde
CAT: Catalase
SOD: Superoxide dismutase
TLR: Toll-like receptors
NF-κB: Nuclear factor kappa-B
MyD88: Myeloiddifferentiationfactor88
JAK: Janus kinase
STAT: Signal transducer and activator of transcription
TNF-α: Tumor necrosis factor alpha
IL-1β: Interleukin 1 beta
TGF-β: Transforming growth factor beta
IFN-γ: Interferon gamma
OUT: Operational taxonomic unit
PCoA: Principal co-ordinates analysis
LEfSe: Line discriminant analysis effect size
SD: Standard deviation
SEM: Standard error of mean
