# Correction: Dietary ellagic acid ameliorated *Clostridium perfringens*-induced subclinical necrotic enteritis in broilers via regulating inflammation and cecal microbiota

**DOI:** 10.1186/s40104-022-00724-0

**Published:** 2022-05-13

**Authors:** Yu Tang, Xinyue Zhang, Yanan Wang, Yongpeng Guo, Peiqi Zhu, Guiguan Li, Jianyun Zhang, Qiugang Ma, Lihong Zhao

**Affiliations:** 1 State Key Laboratory of Animal Nutrition, Poultry Nutrition and Feed Technology Innovation Team, College of Animal Science and Technology, China Agricultural University, No. 2. West Road Yuanming yuan, Beijing, 100193 People’s Republic of China; 2 Jiangsu Lihua animal husbandry Co.,Ltd., No. 500, Hexi Village, Luxi Village Committee, Niutang Town, Wujin District, Changzhou City, Jiangsu Province, 213168 People’s Republic of China; 3 COFCO feed Co.,Ltd., 4th Floor, No. 6, Nandaan Hutong, Xicheng District, Beijing, 100193 People’s Republic of China

**Correction to: J Anim Sci Biotechnol 13, 47 (2022)**

**https://doi.org/10.1186/s40104-022-00694-3**

After publication of the original article [1], it was brought to our attention that the first author Yu Tang is incorrectly listed as one of the corresponding authors, but the article has only one corresponding author: Lihong Zhao.

The original paper has been updated.
