# Supplementary material for: Dietary ellagic acid ameliorated Clostridium perfringens-induced subclinical necrotic enteritis in broilers via regulating inflammation and cecal microbiota
Source: J Anim Sci Biotechnol. 2022 Apr 19;13:47. doi: 10.1186/s40104-022-00694-3 (PMC9016943; doi:10.1186/s40104-022-00694-3)
Supplement: Supplementary file 1 — Additional file 1: Table S1. Composition and nutrient levels of the basal diets. Table S2. Primers used for quantitative real-time PCR. Table S3. Good’s coverage estimators of C. perfringens challenge and dietary EA levels treatments. Table S4. Effect of C. perfringens challenge and dietary EA levels on alpha diversity of cecal microbiota. Fig. S1. Effect of C. perfringens challenge and dietary EA levels on relative mRNA expression of jejunal inflammation-related pathway and cytokine genes in jejunal mucosa of broilers at d 42. (A and B) The relative mRNA expressions of transforming growth factor-β (TGF-β) and interferon γ (IFN-γ). (C) The relative mRNA expression of myeloiddifferentiationfactor88 (MyD88). (D, E and F) The relative mRNA expression of Janus kinase 1 (JAK1), Janus kinase 2 (JAK2), and signal transducers and activators of transcription 1 (STAT1). Unchallenged, birds without C. perfringens infection; challenged, birds with C. perfringens infection. Values are means (n = 6) with their standard errors represented by vertical bars. Fig. S2. The quality of sequencing data and beta diversity of cecal microbiota. (A) Venn diagram of the OTUs. (B) The rarefaction curve analysis of the microbial species. (C and D) Box plot and principal co-ordinates analysis (PCoA) plot of beta diversity. All values are expressed as the means (n = 5 in EAXCP group, n = 6 in the other three groups). Fig.S3. The most abundant (top 10) phyla and genus of cecal microbiota. (A) The most abundant (top 10) phyla of cecal microbiota. (B) The most abundant (top 10) genus of cecal microbiota. (C, D, E, F and G) The relative abundance of Firmicutes, Desulfobacterota, Campilobacterota, Elusimicrobia and Ruminococcus torques group. [file 40104_2022_694_MOESM1_ESM.docx]

**Supplementary materials**

**Table S1** Composition and nutrient levels of the basal diets

| Items, % | d 1-21 | d 22-42 |
| --- | --- | --- |
| Ingredients |  |  |
| Corn | 56.44 | 55.90 |
| Soybean meal(43% CP） | 32.11 | 31.49 |
| Dicalcium phosphate | 1.64 | 1.32 |
| Limestone | 1.40 | 1.32 |
| NaCl | 0.30 | 0.30 |
| DL-Met (98%) | 0.21 | 0.10 |
| Multimineral * | 0.15 | 0.15 |
| Multivitamin † | 0.15 | 0.15 |
| Choline chloride (60%) | 0.10 | 0.10 |
| Soybean oil | 3.16 | 4.94 |
| Phytase (10000 IU/g ) | 0.01 | 0.01 |
| L-Lys-H2SO4 (70%) | 0.11 | 0.00 |
| Zeolite powder | 0.22 | 0.22 |
| corn gluten meal | 4.00 | 4.00 |
| SUM | 100.00 | 100.00 |
|  |  |  |
| Nutrient and energy concentration ‡ |  |  |
| Metabolisable energy, kcal/kg | 2950 | 3050 |
| Crude protein | 21 | 20 |
| Ca | 1.0 | 0.9 |
| P | 0.64 | 0.58 |
| STTD P | 0.46 | 0.42 |
| Lys | 1.1 | 1.0 |
| Met | 0.89 | 0.77 |
| Thr | 0.78 | 0.76 |
| Trp | 0.22 | 0.22 |

* Supplied the following per kg complete feed: Cu, 8 mg; Zn, 75 mg; Fe, 80 mg; Mn, 100 mg; Se, 0.15 mg; I, 0·35 mg.

† Supplied the following per kg complete feed: retinyl acetate, 24 mg; cholecalciferol, 6 mg; menadione, 2·65 mg; thiamine, 2 mg; riboflavin, 6 mg; cyanocobalamin, 0·025 mg; α-tocopheryl acetate, 20 mg; biotin, 0·0325 mg; folic acid, 1·25 mg; pantothenic acid, 12 mg; niacin, 50 mg.

‡ Calculated value.

**Table S2** Primers used for quantitative real-time PCR

| Target genes | Primer sequence (5' to 3') |
| --- | --- |
| *TNF-α* | F:ATGAACCCTCCGCAGTACTC |
|  | R:AAGAGGCCACCACACGACA |
| *IL-1β* | F:CCTGTCTCTGTCCCTACCCCCTA |
|  | R:GTCAACGGGTGTGCTGCAGGAAC |
| *IL-8* | F:CCAAGCACACCTCTCTTCCA |
|  | R:GCAAGGTAGGACGCTGGTAA |
| *TGF-β* | F:ATGTGTTCCGCTTTAACGTGTC |
|  | R:GCTGCTTTGCTATATGCTCATC |
| *IFN-γ* | F:GCTCCCGATGAACGACTTGA |
|  | R:TGTAAGATGCTGAAGAGTTCATTCG |
| *TLR2* | F:GGGGCTCACAGGCAAAATC |
|  | R:AGCAGGGTTCTCAGGTTCACA |
| *TLR4* | F:AGTCTGAAATTGCTGAGCTCAAAT |
|  | R:GCGACGTTAAGCCATGGAAG |
| *MyD88* | F:GAAGTTGGGCCACGACTACCT |
|  | R:TTGCACTTGACCGGAATCAGC |
| *NF-κB* | F:TGACCGCCAATAGCTTGTCC |
|  | R:ACAGCTAAATGCAATGCCGTTC |
| *JAK1* | F:TGCACCGTGACTTAGCAGCAAG |
|  | R:TCTGAATCAAGCATTCTGGAGCATACC |
| *JAK2* | F:TCGCTATGGCATTATTCG |
|  | R:GTGGGGTTTGGTCCTTTT |
| *JAK3* | F:GCATCCGCCGCCGTGTTG |
|  | R:AGCACCGCAGCCTCTCCAG |
| *STAT1* | F:TAAAGAGGGAGCAATCAC |
|  | R:ATCAGGGAAAGTAACAGC |
| *STAT6* | F:GCAACCTCTACCCCAACA |
|  | R:TCCCTTTCGCTTTCCACT |
| *iNOs* | F:TGGGTGGAAGCCGAAATA |
|  | R:GTACCAGCCGTTGAAAGGAC |
| *ZO-1* | F:CTTCAGGTGTTTCTCTTCCTCCTC |
|  | R:CTGTGGTTTCATGGCTGGATC |
| Occludin | F:ACGGCAGCACCTACCTCAA |
|  | R:GGGCGAAGAAGCAGATGAG |
| Claudin-2 | F:CTGCTCACCCTCATTGGA |
|  | R:AACTCACTCTTGGGCTTCTG |
| β-actin | F:AGACATCAGGGTGTGATGGTTGGT |
|  | R:TGGTGACAATACCGTGTTCAATGG |

*F*, forward; *R*, reverse. *TNF-α*, tumor necrosis factor alpha; *IL-1β*, interleukin 1 beta; *IL-8*, interleukin 8; *TGF-β*, transforming growth factor beta; *IFN-γ*, interferon gamma; *TLR-2*, toll-like receptor 2; *TLR-4*, toll-like receptor 4; *MyD88*, myeloiddifferentiationfactor88; *NF-κB*, nuclear factor kappa B; *JAK1*, Janus kinase 1; *JAK2*, Janus kinase 2; *JAK3*, janus kinase 3; *STAT1*, signal transducers and activators of transcription 1; *STAT6*, signal transducers and activators of transcription 6; *iNOS*, inducible nitric oxide synthase; *ZO-1*, zonula occludens 1.

**Table S3** Good's coverage estimators of *C. perfringens* challenge and dietary EA levels treatments

| Dietary EA levels | 0 mg/kg | | 500 mg/kg | |  |
| --- | --- | --- | --- | --- | --- |
| *C. perfringens* challenge | - | + | - | + | SEM |
| Good's coverage | 0.9976 | 0.9980 | 0.9976 | 0.9979 | 0.00007766 |

All values are expressed as the means (*n* = 5 in EAXCP group, *n* = 6 in the other three groups).

**Table S4** Effect of *C. perfringens* challenge and dietary EA levels on alpha diversity of cecal microbiota

| Dietary EA levels | 0 mg/kg | | 500 mg/kg | |  | *P*-values | | |
| --- | --- | --- | --- | --- | --- | --- | --- | --- |
| *C. perfringens* challenge | - | + | - | + | SEM | *C. perfringens* challenge | Dietary EA level | Interaction |
| Observed species | 715.33 | 728.33 | 780.00 | 762.20 | 12.052 | 0.9182 | 0.0459* | 0.5123 |
| Shannon | 5.77 | 5.87 | 5.96 | 6.39 | 0.099 | 0.2241 | 0.0465* | 0.4608 |
| Chao1 | 1200.58 | 987.58 | 1043.61 | 996.84 | 65.301 | 0.3489 | 0.5914 | 0.5462 |
| Simpson | 0.94 | 0.94 | 0.93 | 0.96 | 0.005 | 0.1841 | 0.4753 | 0.0836 |
| ACE | 813.03 | 802.70 | 870.52 | 836.73 | 14.425 | 0.4505 | 0.1265 | 0.6866 |

All values are expressed as the means (*n* = 5 in EAXCP group, *n* = 6 in the other three groups).* Significant main effect (*P* < 0.05) of *C. perfringens* challenge or dietary EA level.


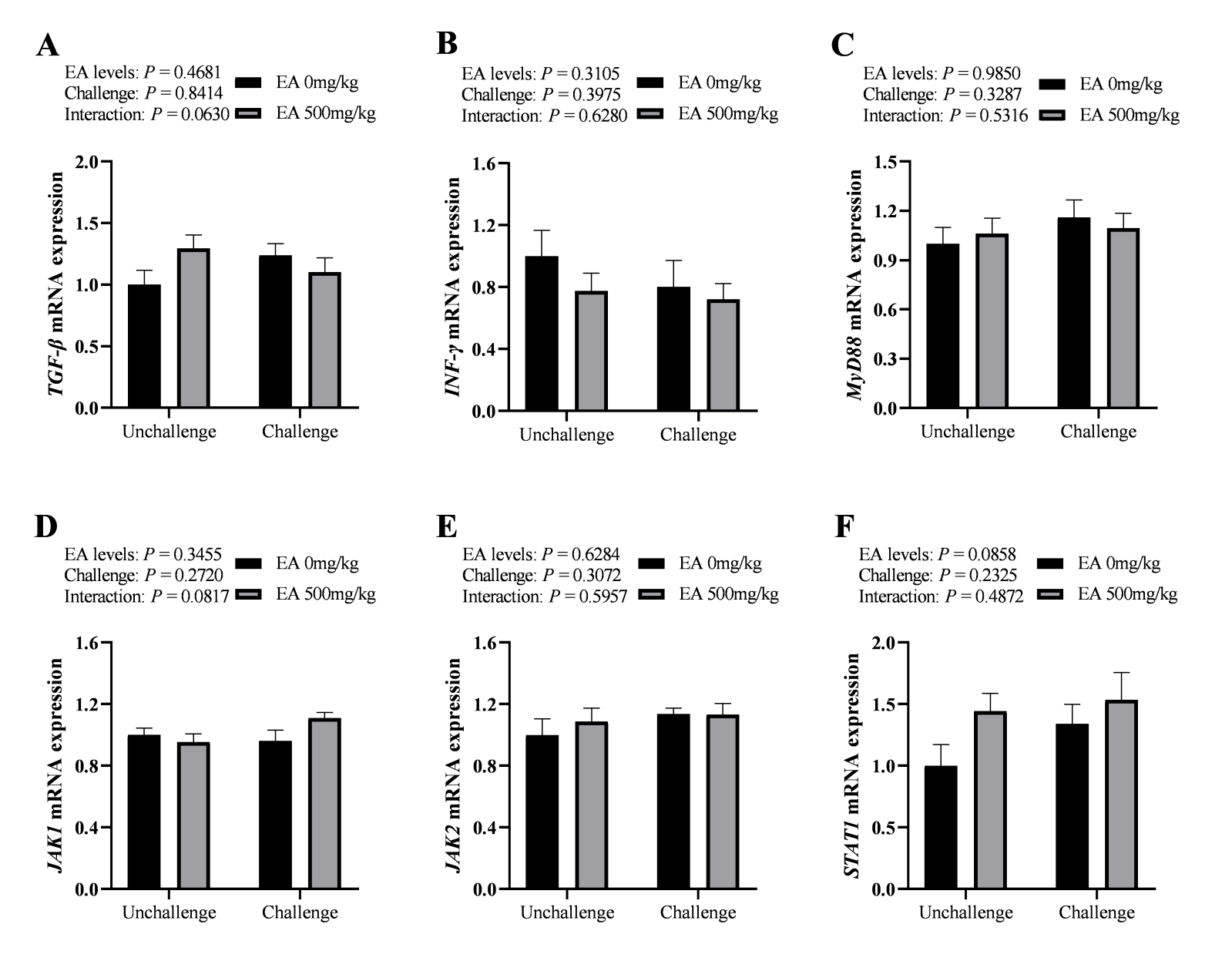


**Fig. S1** Effect of *C. perfringens* challenge and dietary EA levels on relative mRNA expression of jejunal inflammation-related pathway and cytokine genes in jejunal mucosa of broilers at d 42. (**A** and **B**) The relative mRNA expressions of transforming growth factor-β (*TGF-β*) and interferon γ (*IFN-γ*). (**C**) The relative mRNA expression of myeloiddifferentiationfactor88 (*MyD88*). (**D**, **E** and **F)** The relative mRNA expression of Janus kinase 1 (*JAK1*), Janus kinase 2 (*JAK2*), and signal transducers and activators of transcription 1 (*STAT1*). Unchallenged, birds without *C. perfringens* infection; challenged, birds with *C. perfringens* infection. Values are means (*n* = 6) with their standard errors represented by vertical bars.


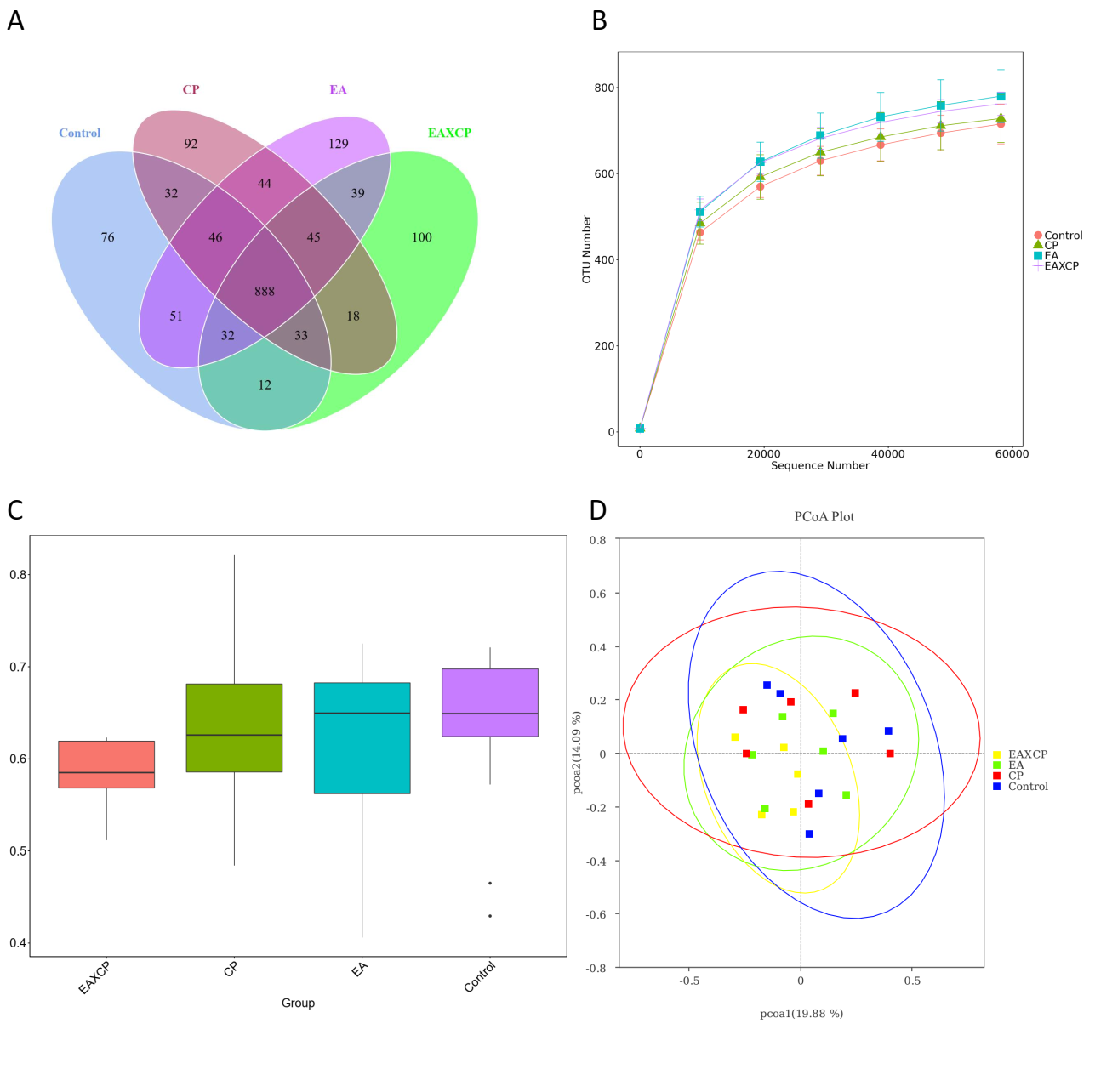


**Fig. S2** The quality of sequencing data and beta diversity of cecal microbiota. (**A**) Venn diagram of the OTUs. (**B**) The rarefaction curve analysis of the microbial species. (**C** and **D**) Box plot and principal co-ordinates analysis (PCoA) plot of beta diversity. All values are expressed as the means (*n*=5 in EAXCP group, *n* = 6 in the other three groups).


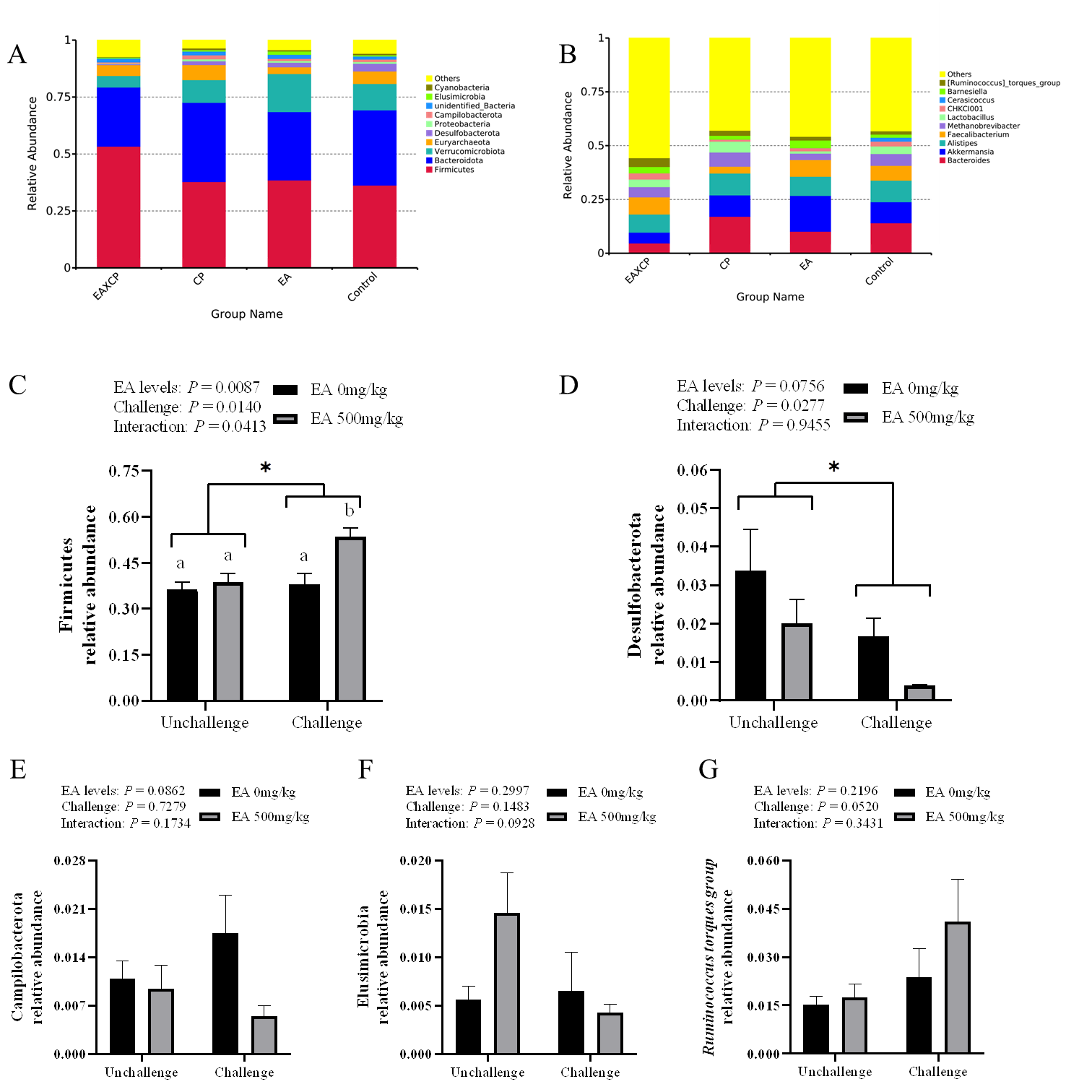


**Fig. S3** The most abundant (top 10) phyla and genus of cecal microbiota. (**A**) The most abundant (top 10) phyla of cecal microbiota. (**B**) The most abundant (top 10) genus of cecal microbiota. (**C**, **D**, **E**, **F** and **G**) The relative abundance of Firmicutes, Desulfobacterota, Campilobacterota, Elusimicrobia and *Ruminococcus torques group*.
